# An integrated omics approach highlights how epigenetic events can explain and predict response to neoadjuvant chemotherapy and bevacizumab in breast cancer

**DOI:** 10.1002/1878-0261.13656

**Published:** 2024-04-26

**Authors:** Thomas Fleischer, Mads Haugland Haugen, Jørgen Ankill, Laxmi Silwal‐Pandit, Anne‐Lise Børresen‐Dale, Ingrid Hedenfalk, Thomas Hatschek, Jörg Tost, Olav Engebraaten, Vessela N. Kristensen

**Affiliations:** ^1^ Department of Cancer Genetics, Institute for Cancer Research Oslo University Hospital Oslo Norway; ^2^ Department of Tumor Biology, Institute for Cancer Research Oslo University Hospital Oslo Norway; ^3^ Institute of Clinical Medicine, Faculty of Medicine University of Oslo Oslo Norway; ^4^ Division of Oncology, Department of Clinical Sciences Lund Lund University Lund Sweden; ^5^ Breast Cancer Center Karolinska University Hospital Stockholm Sweden; ^6^ Department of Oncology‐Pathology Karolinska Institutet Stockholm Sweden; ^7^ Laboratory for Epigenetics and Environment, Centre National de Recherche en Génomique Humaine, CEA – Institut de Biologie François Jacob Université Paris Saclay Evry France; ^8^ Division of Cancer Medicine, Department of Oncology Oslo University Hospital Oslo Norway; ^9^ Department of Medical Genetics Oslo University Hospital Oslo Norway

**Keywords:** bevacizumab, breast cancer, chemotherapy, DNA methylation, epigenetics, multiomics

## Abstract

Treatment with the anti‐angiogenic drug bevacizumab in addition to chemotherapy has shown efficacy for breast cancer in some clinical trials, but better biomarkers are needed to optimally select patients for treatment. Here, we present an omics approach where DNA methylation profiles are integrated with gene expression and results from proteomic data in breast cancer patients to predict response to therapy and pinpoint response‐related epigenetic events. Fresh‐frozen tumor biopsies taken before, during, and after treatment from human epidermal growth factor receptor 2 negative non‐metastatic patients receiving neoadjuvant chemotherapy with or without bevacizumab were subjected to molecular profiling. Here, we report that DNA methylation at enhancer CpGs related to cell cycle regulation can predict response to chemotherapy and bevacizumab for the estrogen receptor positive subset of patients (AUC = 0.874), and we validated this observation in an independent patient cohort with a similar treatment regimen (AUC = 0.762). Combining the DNA methylation scores with the scores from a previously published protein signature resulted in a slight increase in the prediction performance (AUC = 0.784). We also show that tumors receiving the combination treatment underwent more extensive epigenetic alterations. Finally, we performed an integrative expression–methylation quantitative trait loci analysis on alterations in DNA methylation and gene expression levels, showing that the epigenetic alterations that occur during treatment are different between responders and non‐responders and that these differences may be explained by the proliferation–epithelial‐to‐mesenchymal transition axis through the activity of grainyhead like transcription factor 2. Using tumor purity computed from copy number data, we developed a method for estimating cancer cell‐specific methylation to confirm that the association to response reflects DNA methylation in cancer cells. Taken together, these results support the potential for clinical benefit of the addition of bevacizumab to chemotherapy when administered to the correct patients.

## Graphical abstract

DNA methylation and machine learning (LASSO leave‐one‐out) was used to identify and validate a signature to identify patients responding to chemotherapy and bevacizumab (pCR: pathological complete response). Further, by integrating DNA methylation and gene expression, we identified alterations in DNA methylation of enhancer CpGs in GRHL2‐binding regions, which explained response.

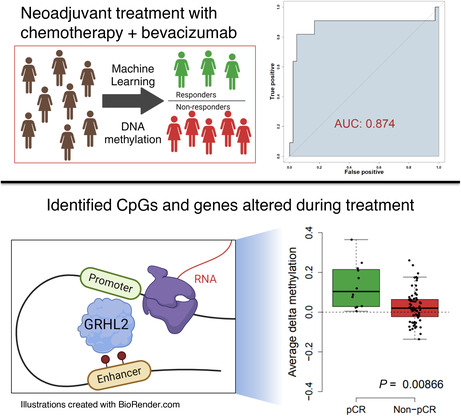

**Abbreviations**
5‐FU: 5‐fluorouracil
ASCAT: allele‐specific copy number analysis of tumors
AUC: area under the curve
ChIA‐PET: chromatin interaction analysis by paired‐end tag sequencing
CNA: copy number alteration
emQTL: expression‐methylation quantitative trait loci
EMT: epithelial‐to‐mesenchymal transition
ER: estrogen receptor
FEC: 5‐fluorouracil, epirubicin, and cyclophosphamide
GEO: Gene Expression Omnibus
GO: gene ontology
GRHL2: grainyhead like transcription factor 2
HER2: human epidermal growth factor receptor 2
IQR: inter‐quartile range
LOO: leave‐one‐out
MSigDB: Molecular Signatures Database
pCR: pathological complete response
PFS: progression‐free survival
RCB: residual cancer burden
RFS: relapse‐free survival
ROC: receiver operating characteristic
TF: transcription factor
TNBC: triple‐negative breast cancer
VEGF: vascular endothelial growth factor

## 1 Introduction

Neoadjuvant treatment is commonly used for patients with large breast tumors to avoid delays in important systemic therapy, to evaluate the efficacy of such therapy and facilitate the surgical resection of breast tumors. With the rationale of denying tumors access to nutrients and oxygen through vascular endothelial growth, anti‐angiogenic therapy has been applied to decrease tumor size in the neoadjuvant setting [1, 2, 3]. Bevacizumab is a monoclonal antibody that binds to vascular endothelial growth factor (VEGF) and blocks the binding to its receptors thus inhibiting endothelial growth [4]. Although the drug has shown efficacy in some trials [5, 6, 7, 8, 9, 10], it did not increase overall survival. We have previously shown that bevacizumab can improve response to neoadjuvant chemotherapy in patients with estrogen receptor (ER)‐positive tumors [11]. Despite favorable clinical effects in many patients, numerous therapies are currently not administered due to the lack of adequate predictive biomarkers; an example in breast cancer is the addition of bevacizumab to chemotherapy. Such treatment modality is today infrequently used despite subgroups of patients responding well. There is accordingly an urgent need for the introduction of novel biomarkers, and a better understanding of the biological mechanisms leading to response or resistance to this treatment. A large meta‐analysis analyzing 2268 patients treated with bevacizumab and 2278 treated without bevacizumab concluded that “Optimizing patient selection is desirable for maximizing the long‐term benefits of [bevacizumab], while reducing cost and treatment‐related adverse effects” [12]. Recently, a signature consisting of the expression of nine proteins was shown to predict response to the combination of chemotherapy and bevacizumab in the NeoAva trial (human epidermal growth factor receptor 2 (HER2) negative, non‐metastatic) with an area under the ROC curve (AUC) of 0.92 for ER positive and 0.85 for all patients [13]. A methylation signature, predictive of response to chemotherapy and bevacizumab has been reported with an AUC of 0.91 in a study with patients with metastatic triple‐negative breast tumors (TNBC) [14], and a hypoxia gene score has been associated with response to chemotherapy and bevacizumab in patients with HER2 negative non‐metastatic disease (odds ratio = 2.4) [15]. Together, these reports highlight the potential for use of molecular signatures for prediction of response.

To study the biological significance of epigenetic changes, we have previously integrated DNA methylation and mRNA expression using an expression‐methylation quantitative trait loci (emQTL) analysis. The emQTL approach enabled us to identify variation in DNA methylation with concomitant variation in gene expression in breast tumors. This led to the discovery of a mechanism to maintain continuously active estrogen receptor signaling in ER‐positive breast tumors through loss of DNA methylation at enhancers carrying binding sites for the transcription factors (TFs) ER, FOXA1 and GATA3 [16], and loss of methylation at enhancers carrying binding sites of TFs related to proliferation in ER negative tumors [17]. We have also shown that DNA methylation of cell cycle genes changes during treatment with chemotherapy (Doxorubicin or Fluorouracil (5FU) and mitomycin C), and that these changes differ between responders and non‐responders [18]. Also, in a study of TNBC receiving neoadjuvant chemotherapy alone, a set of differentially methylated regions at diagnosis was associated to response and overall survival [19]. Treatment‐induced epigenetic changes at enhancers through DNA methylation have to our knowledge not yet been investigated. There are several potential advantages of performing the analysis using DNA methylation data as these data may reveal information related to cell identity and tumor origin. Furthermore, different 'omics data contain complementary information, and multi‐omic biomarkers may thus be more reliable.

In the current study, we aimed at developing a predictive signature based on DNA methylation that could assist in selecting patients for the combination therapy of chemotherapy and bevacizumab. We also investigated alterations in DNA methylation that were both common to all patients as well as patient‐specific and could explain varying response to treatment.

## 2 Materials and methods

### 2.1 Study design and patient material

The NeoAva clinical trial has been reported previously [11]. In brief, patients were recruited at Oslo University Hospital and St. Olavs hospital (Trondheim) between 2008 and 2012, and written informed consent was obtained from all patients prior to inclusion. The study was approved by the Institutional Protocol Review Board of Oslo University Hospital, the Regional Committee for Medical and Health Research Ethics for South‐Eastern Norway (ref. no. 2008/10187), and the Norwegian Medicines Agency, and carried out in accordance with the Declaration of Helsinki, International Conference on Harmony/Good Clinical practice. The study is registered in the ClinicalTrials database with the identifier NCT00773695.

Patients with HER2‐negative mammary carcinomas with size > 2.5 cm previously untreated for breast cancer and no sign of metastatic disease were eligible. The patients were randomized 1 : 1 to receive bevacizumab and chemotherapy (combination treatment arm) or chemotherapy alone (chemotherapy arm). Eighty‐two of 132 patients were screened for germline BRCA1/2 mutations in separate blood samples using sequencing or the Decaplex panel; of these, two patients were found to be mutation carriers.

The chemotherapy regimen consisted of four cycles of 5‐fluorouracil 600 mg·m^−2^, epirubicin 100 mg·m^−2^, and cyclophosphamide 600 mg·m^−2^ (FEC) every 3 weeks, followed by docetaxel 100 mg·m^−2^ every 3 weeks or 12 weekly infusions of paclitaxel 80 mg·m^−2^. Bevacizumab was administered intravenously at a dose of 15 mg·kg^−1^ every third week or 10 mg·kg^−1^ every other week in patients receiving docetaxel or paclitaxel, respectively. Tumor biopsies were sequentially collected before treatment (core needle biopsies; termed “week 0” samples), during treatment (core needle biopsies 12 weeks into treatment; termed “week 12” samples), and after treatment (at surgery; termed “week 25” samples) and immediately transferred to −80°C. The needle biopsies had a mean weight of 33 mg (range 6.0–80.5 mg) and the surgical specimen at 25 weeks had a mean weight of 228 mg (range 28.0–685 mg). Response to treatment was measured as either pathologic complete response (pCR; complete eradication of all invasive cancer cells in both breast and axillary lymph nodes), residual cancer burden (RCB), or as fraction of tumor volume left after treatment. RCB was calculated using the RCB Calculator [20, 21], dichotomized to low and high for RCB 0/I and RCB II/III, respectively. Continuous response was determined as the tumor volume left at surgery relative to pre‐treatment as measured by MRI, ultrasound or pathological inspection (MRI was preferred when available). The patient characteristics for all patients are shown in Table S1A, and the characteristics for patients in the combination arm (separated by pCR or residual disease) are shown in Table S1B.

### 2.2 DNA extraction and DNA methylation profiling

Fresh‐frozen tumor biopsies were dissected into small pieces, mixed, and divided into amounts suitable for DNA, RNA, and protein extraction. DNA was isolated using the QIAcube and AllPrep DNA/RNA Mini Kit (Qiagen, Hilden, Germany) 350 or 600 for biopsies from the first two or the last time point, respectively (Qiagen) according to the company's standard protocol. The DNA methylation status of more than 450 000 CpG sites was interrogated using the Infinium (Illumina, Inc., San Diego, CA, USA) Preprocessing and normalization involved steps of probe filtering, color bias correction, background subtraction and subset quantile normalization as previously described [22]. Raw and normalized data are available from the Gene Expression Omnibus (GEO) with accession number GSE207460.

### 2.3 mRNA and copy number profiling

Gene expression profiling has been previously reported [11]. Briefly, the analysis was performed using one color SurePrint G3 Human GE 8x60k Microarrays (Agilent Technologies, Santa Clara, CA, USA), and the data is available in the ArrayExpress database (http://www.ebi.ac.uk/arrayexpress) under accession number E‐MTAB‐4439. The PAM50 subtyping of breast cancer divides breast tumors into five subtypes: Luminal A, Luminal B, HER2 enriched, Basal‐like and Normal‐like. Luminal tumors are most often ER positive with Luminal B having higher proliferation; HER2 enriched are either ERBB2/HER2 amplified or have higher expression of this pathway; Basal‐like have no overexpressed receptors and harbor widespread copy number alterations (CNAs); and Normal‐like have expression profiles similar to normal breast tissue [23, 24]. The Parker algorithm [25] was used to determine the molecular subtype of each sample.

Copy number alteration profiles have previously been reported [26]. Briefly, tumor DNA was analyzed for CNAs using Genome‐Wide Human SNP array 6.0 (Affymetrix, Santa Clara, CA, USA). The copy number profiles were segmented with the allele‐specific piecewise constant fitting algorithm [27], and subsequently, allele‐specific copy number analysis of tumors (ASCAT [28]) was used to estimate tumor cell fraction, tumor ploidy, and copy number. The data is deposited at the European Genome‐phenome Archive (https://ega‐archive.org/) under accession number EGAS00001003287.

### 2.4 Validation data

Validation for the predictive signature for response to chemotherapy and bevacizumab was performed in the PROMIX trial (*N* = 150; inclusion between 2008 and 2011; ClinicalTrials.gov NCT00957125 [29]), a single arm phase II neoadjuvant trial where patients with HER2 negative tumors received chemotherapy in combination with bevacizumab. The chemotherapy regimen consisted of six cycles of epirubicin combined with docetaxel. The patient characteristics of the PROMIX trial were similar to NeoAva (Table S1A,C). Gene expression data is available through GEO with accession number GSE87455. DNA methylation data was not available for this patient cohort. For validation of the predictive signature reported here, only ER‐positive patients of the PROMIX trial were used.

Validation for the concomitant alterations in DNA methylation and gene expression was performed in the Doxo/Fumi data set. The Doxo cohort consists of 93 breast cancer patients (median age was 64 years; range, 32–88 years) that were treated with doxorubicin monotherapy in a neoadjuvant setting [30]. The Fumi cohort consists of 35 breast cancer patients (median age was 67 years; range 37–82 years) that were treated with 5‐fluorouracil and mitomycin in a neoadjuvant setting [31]. Patients in both cohorts had locally advanced disease (T3/T4 and/or N2 tumors), and were included between 1991 and 2001. Because of their similar patient characteristics, the Doxo and Fumi cohorts have been combined and analyzed together in previous studies [18, 32]. In the present study, DNA methylation was available for tumor samples taken before and after treatment from both cohorts (Doxo/Fumi).

### 2.5 Statistical analyses

All statistical and bioinformatical analyses were carried out in the r software version 4.1.2 [33] unless otherwise specified.

### 2.6 Prediction of response to combination treatment

Prediction of patient achieving pCR from DNA methylation data was performed using the machine learning method LASSO [34]. This method is susceptible to overfitting if the number of features is too large and if there are features with no biological importance. We thus performed a preselection of CpGs, restricting the data to include only CpGs indicated to be involved in epigenetic regulation of processes considered important in primary breast tumors based on our recently reported improved emQTL analysis [17]. We identified 44 263 CpGs involved in four mechanisms related to breast tumor biology. A CpG was selected when either involved in (a) estrogen‐regulated proliferation in tumor cells, (b) non‐estrogen‐regulated proliferation in tumor cells, (c) varying immune infiltration, or (d) varying fibroblast infiltration. These 44 263 CpGs were used in the LASSO analysis.

We constructed Lasso models using the r package glmnet [35] in an internal validation strategy (leave‐one‐out; LOO) where models are trained on *N* − 1 samples (where *N* is the total number of samples in the analysis) and applied to the one remaining sample. This is run *N* times to construct *N* models. To generate the common DNA methylation signature, we include the CpGs present in at least 50% of the models, and average the model coefficients. Further, to generate a DNA methylation score for each patient, we multiply the model coefficients (either from the LOO models or the common DNA methylation signature) with the associated methylation value, and sum across the CpGs included in the model. The score can be retrieved either using the r function *predict*() or calculated manually. Transforming this score to values between 0 and 1 represents the probability of achieving pCR. To avoid selection bias we also performed nested cross‐validation (CV; function *cv.glmnet*), where another 5‐fold CV was applied within each sub‐training set to determine the optimal model parameters, e.g., the weight and beta values of the Lasso model. The other model parameters were set to their default values. Receiver operating characteristic (ROC) curves were generated using the proc r package [36].

To translate a DNA methylation signature to a gene expression signature, we selected the genes whose expression had the highest absolute correlation to the CpGs in the signature. The beta coefficients of the linear model to be applied on gene expression data were determined by multiplying the beta coefficients for each CpG of the linear model of DNA methylation with the correlation coefficients (rho values) between DNA methylation and gene expression.

For comparison, the approach was also performed directly on gene expression data using the same preselection strategy; 4904 genes correlating to the DNA methylation positions and involved in the same four biological mechanisms [17] were pre‐selected for the analysis.

### 2.7 Hybrid protein‐methylation prediction score

To compare and improve the performance of the DNA methylation‐based and a protein‐based prediction of response to the combination therapy, we generated a hybrid score integrating the previously published nine‐protein ViRP signature [13] and the newly discovered DNA methylation signature. In the validation data set (PROMIX [29]), both signatures were translated to mRNA signatures, and the signatures were applied to generate ViRP scores and DNA methylation scores. Then, the scores were standardized (mean = 0; SD = 1), and the average value was calculated for each patient.

### 2.8 Identification of treatment‐induced changes in DNA methylation

To assess treatment‐induced changes in DNA methylation, we performed paired *t*‐tests (r function *t.test*) between week 0 and week 12 samples, between week 12 and week 25 samples, as well as between week 0 and week 25 samples. CpGs were included in the analysis if the inter‐quartile range (IQR) was larger than 0.1. Alterations were considered significantly different if the Benjamini–Hochberg corrected *P*‐value was smaller than 0.05 and the absolute change in DNA methylation was larger than 0.1.

### 2.9 Identification of concomitant changes in DNA methylation and gene expression (delta emQTL)

To identify epigenetic alterations with concomitant changes in genes expression, potentially highlighting transcriptional programs under epigenetic control that are altered during treatment, we performed an emQTL analysis [16, 17] using delta methylation and delta expression levels (changes between week 0 and week 12). Correlation analyses were performed between all possible CpG‐gene pairs, and correlations were considered significant if the Bonferroni‐corrected *P*‐value was less than 0.05. Bonferroni correction of *P*‐values in this analysis was chosen to conform to previous reports [16, 17].

For all CpGs and genes with at least one significant correlation, spectral co‐clustering (biclustering) was performed on the inverse correlation coefficients using the *SpectralCoclustering* algorithm (random state = 0) contained within the scikit‐learn library [37] in python version 3.7.9.

### 2.10 Gene set enrichment analysis and hierarchical clustering

Gene sets used for gene set enrichment analysis were downloaded from the Molecular Signatures Database v7.4 (MSigDB) [38]. Enrichment was determined by hypergeometric testing (r function *phyper*) using the Hallmark (H) and gene ontology (GO; C5) gene set collections. *P*‐values were corrected for multiple testing using Benjamini–Hochberg correction (r function *p.adjust*).

Hierarchical clustering of the DNA methylation and gene expression levels was performed using the r package *pheatmap* [39] using Euclidean distance and ward.D2 linkage.

### 2.11 Genomic segmentation and annotation

ChromHMM segmentation data from cell lines representing different breast cancer subtypes were obtained from Xi et al. [40], and included MCF7 and ZR751 (Luminal A), UACC812 and MB361 (Luminal B), HCC1954 and AU565 (HER2+), HCC1937 and MB469 (Basal‐like). In this work, ChIP‐seq peaks from key histone modifications including H3K4me3, H3K4me1, H3K27me3, H3K9me3, and H3K36me3 were used to predict chromatin states across the genome of the cell lines. The genomes were annotated into 13 distinct chromatin states including active promoter (PrAct), active promoter flanking (PrFlk), active transcription (TxAct), active transcription flanking (TxFlk), active intergenic enhancer (EhAct), active genic enhancer (EhGen), bivalent promoter (PrBiv), bivalent enhancer (EhBiv), repressive polycomb domain (RepPC), weak repressive domain (WkRep), repeat/ZNF genes (RpZNF), heterochromatin (Htchr) and quiescent state/low signals (QsLow) [40]. Subtype specific ChromHMM annotations were made by collapsing the ChromHMM annotations from cell lines of similar subtype, omitting genomic regions with discordant annotation within a subtype. Enrichment of CpGs in a ChromHMM defined functional region was measured as the ratio between the frequency of delta emQTL CpGs found in a specific segment type over the frequency of CpGs from the Illumina HumanMethylation450 array found within the same segment type. *P*‐values were obtained by hypergeometric testing with the Illumina 450k array probes as background (*n* = 485 512). *P*‐values were corrected for multiple testing using Benjamini–Hochberg procedure.

### 2.12 TF enrichment analysis in UniBind defined TF binding sites

Enrichment of CpGs in TF binding sites was assessed using data obtained from the UniBind database [41]. UniBind is a database for predicted direct TF‐DNA interactions by combining motif information with ChIP‐seq data from 1983 ChIP‐seq experiments for 231 unique human TFs from 315 different cell lines and tissues. Maps of direct TF‐DNA interactions were downloaded from the UniBind website (https://unibind2018.uio.no). The genomic positions of all CpGs from the Illumina 450k array lifted over from hg19 to hg38 using the LiftOver webtool from UCSC genome browser (https://genome.ucsc.edu) and extended with 150 bp upstream and downstream. Since each TF can have multiple TF binding sites derived from different ChIP‐seq experiments, we merged the TF binding sites for all ChIP‐seq experiments for each TF; this analysis is therefore not specific for breast cancer tissue. Enrichment of CpGs in TF binding sites was computed using hypergeometric testing with IlluminaMethylation450 Bead Chip CpGs as background. False discovery rate was estimated by the Benjamini–Hochberg correction.

### 2.13 Chromatin interaction mapping

Chromatin Interaction Analysis by Paired‐End Tag sequencing (ChIA‐PET) is a method to identify promoter‐enhancer loops on a genome‐wide scale [42]. ChIA‐PET data defining long‐range chromatin interactions in the ER+ MCF7 breast cancer cell line was obtained from ENCODE (Accession number ENCR000CAA [42]). Only *cis* loops were included in the analysis. An emQTL was considered to be in a ChIA‐PET Pol2 loop if the CpG and transcription start site of its associated gene were found within the genomic intervals of two opposite ends of the same loop. Enrichment of *in cis* (i.e. on the same chromosome) emQTLs in ChIA‐PET loops was determined by hypergeometric tests using all possible *in cis* CpG‐gene pairs as background.

### 2.14 Generation of tumor purity‐corrected DNA methylation profiles

To determine cancer cell‐specific alterations in DNA methylation, we developed a method to estimate the cancer cell‐specific methylation level in biopsies with varying tumor purity. To achieve this, we utilized ASCAT‐estimated tumor purity [28] generated from CNA measurements in the same tumors [26]. For each CpG, a linear regression was constructed between the ASCAT‐estimated tumor purity and measured DNA methylation. The methylation level for the regression line at tumor purity 100% was considered the mean tumor cell methylation, and each sample's distance to the regression line (residual) added to the mean tumor cell methylation was made to represent the tumor methylation. Consequently, each CpG was adjusted independently, depending on its association to tumor purity. This approach allowed us to analyze two versions of the DNA methylation data: original DNA methylation and tumor purity‐adjusted DNA methylation.

## 3 Results

### 3.1 DNA methylation in pre‐treatment primary tumors predicts response to combination therapy with chemotherapy and bevacizumab

**Fig. 1:**
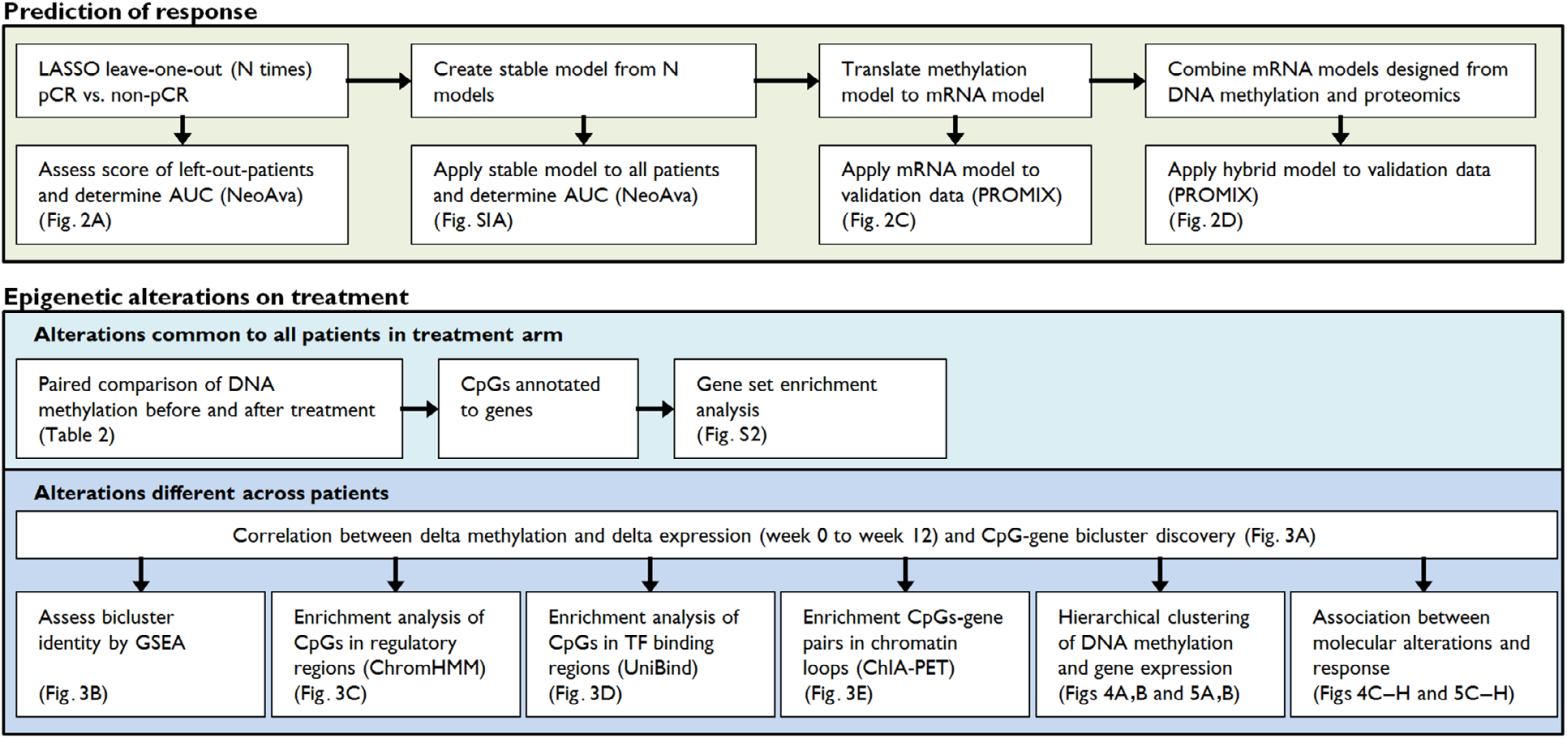
Flow chart of analyses indicating key results. The analyses were split in two main parts: prediction of response and epigenetic alterations during treatment. AUC, area under the curve; ChIA‐PET, chromatin Interaction Analysis by Paired‐End Tag sequencing; GSEA, gene set enrichment analysis; pCR, pathological complete response; TF, transcription factor.

DNA methylation profiling was performed on fresh‐frozen core biopsies from breast tumors from patients receiving neoadjuvant chemotherapy with or without bevacizumab. Biopsies were taken before treatment (week 0), after FEC treatment (weeks 12), and surgical specimens after taxane treatment (week 25). A flow chart of the performed analyses is shown in Fig. 1.

**Fig. 2:**
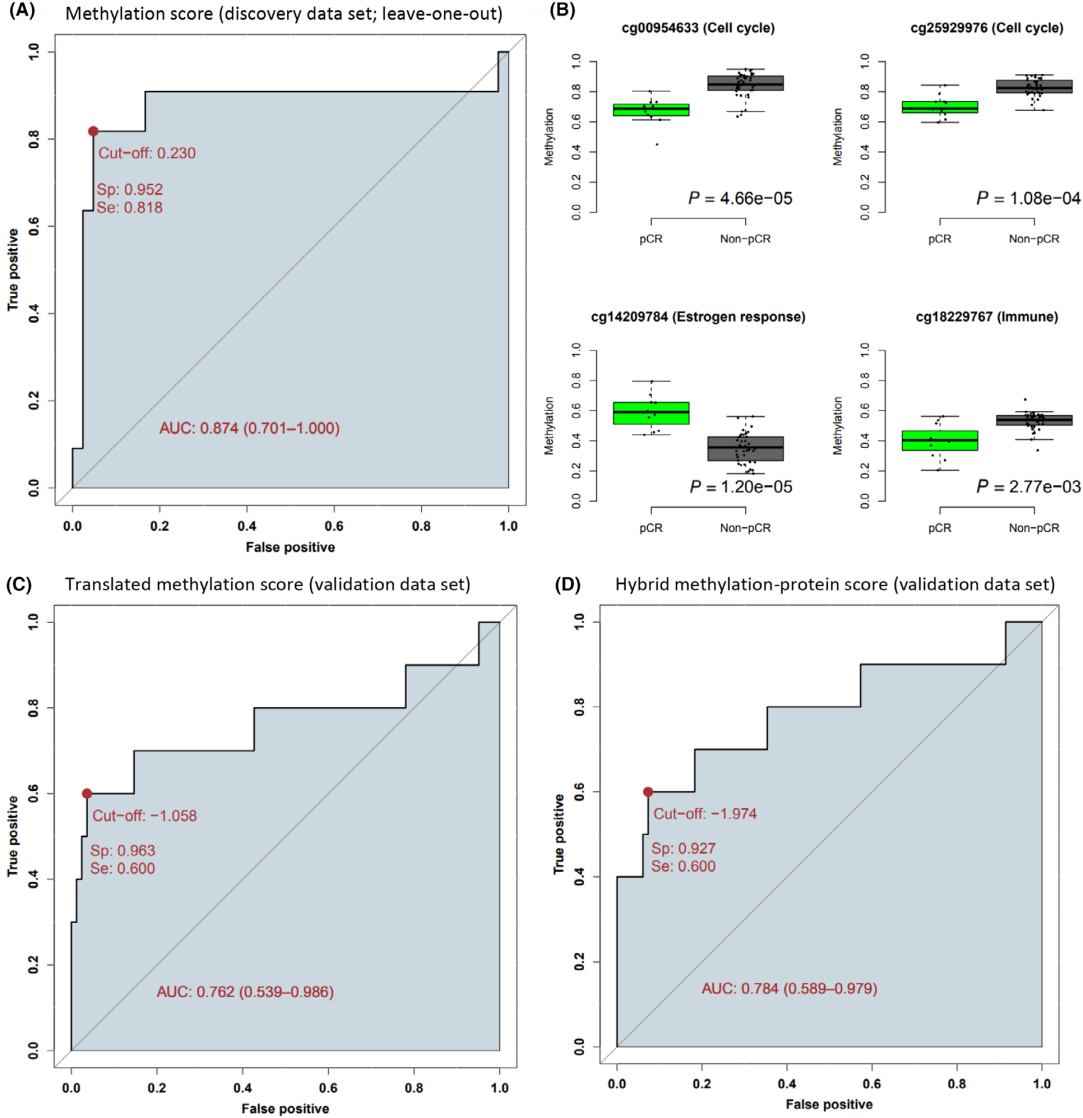
Identification of predictive signatures for response to neoadjuvant treatment with chemotherapy plus bevacizumab in ER‐positive breast cancer. (A) Receiver operating characteristic (ROC) curve showing specificity vs sensitivity for the leave‐one‐out cross‐validation probabilities for response. (B) Boxplots of DNA methylation levels in pre‐treated samples for example CpGs in the identified predictive signature. *P*‐value is determined using a two‐sided *t*‐test. (C, D) ROC curves showing specificity vs sensitivity in the PROMIX validation cohort (C) the “translated” gene expression signature, and (D) the hybrid protein‐methylation score. AUC, area under the curve; Se, sensitivity; Sp, specificity.

To assess the potential of DNA methylation to predict treatment response in pre‐treatment tumor samples, we performed a machine learning approach (LASSO) to identify the CpGs most predictive of pCR. 44 263 CpGs were pre‐selected for the analysis (see Section 2). To produce a predictive signature and avoid overfitting, we performed an internal validation approach (leave‐one‐out cross‐validation) and executed the analysis on each treatment arm individually (to avoid mixing the response to two different treatment regimens). Further, the analysis was performed on ER‐positive and ER‐negative samples separately, as well as independently of ER status. For patients in the combination arm with ER‐positive tumors and available DNA methylation profiles (*n* = 53), the leave‐one‐out approach was able to identify patients who would achieve pCR using pre‐treatment samples, giving an area under the ROC curve of 0.874 (Fig. 2A). After selection of an optimal cutoff, we obtained a sensitivity of 82% and a specificity of 95%. To obtain a stable DNA methylation signature, we combined the 53 models by averaging the linear regression beta coefficients for CpGs present in more than 50% of the models yielding an 11 CpG signature (Table 1). This signature was applied to the DNA methylation data generating a DNA methylation score, perfectly predicting patients achieving pCR (area under the ROC curve of 1; Fig. S1A).

**Table 1:** Predictive signatures for response to neoadjuvant treatment with chemotherapy plus bevacizumab in ER‐positive breast cancer. Aggregate predictive signature for response to therapy. Beta methylation is the linear regression coefficient for DNA methylation, Gene is the gene with highest absolute correlation to the CpG, Correlation meth‐expr is the Pearson correlation coefficient between DNA methylation and gene expression, Beta expression is the “translated” linear regression coefficient (i.e., Beta methylation multiplied by Correlation meth‐expr), emQTL cluster is the previously identified biological function of the CpG.

Probe	Beta methylation	Gene	Correlation meth‐expr	Beta expression	emQTL cluster
cg00954633	−0.42	*PALM*	0.64	−0.27	Cell cycle
cg25929976	−0.20	*CTSC*	−0.52	0.10	Cell cycle
cg19901005	−0.18	*SH2D2A*	−0.58	0.10	Cell cycle
cg12445422	−0.16	*YBX1*	−0.67	0.10	Cell cycle
cg16004056	−0.09	*TBX19*	−0.68	0.06	Cell cycle
cg12772314	−0.07	*ATP6AP1L*	0.41	−0.03	Cell cycle
cg14209784	0.97	*TNFAIP3*	0.64	0.62	Estrogen response 1
cg05870586	0.39	*GALNT10*	−0.56	−0.22	Estrogen response 1
cg08287887	0.08	*JMJD8*	−0.60	−0.05	Estrogen response 1
cg18229767	−0.09	*TNFSF14*	−0.60	0.06	Immune
cg09524658	−0.08	*MLKL*	−0.62	0.05	Immune

The majority of the CpGs in the predictive pre‐treatment signature were associated with cell cycle regulation, three CpGs were associated with estrogen response, and two CpGs were associated with varying immune infiltration. The CpGs were significantly differently methylated between responders and non‐responders (examples in Fig. 2B), with CpGs involved in cell cycle regulation and immune infiltration being less methylated in responders, and CpGs involved in estrogen response being more methylated in responders.

The fact that the pre‐selected CpGs were strongly associated to expression levels of genes [17] enabled us to calculate a “translated” signature by multiplying the methylation beta coefficients with the correlation coefficients between the methylation of a CpG and expression of the gene with highest absolute correlation. In this way, we are leveraging on the high correlation between DNA methylation and gene expression to construct a predictive signature that could be applied also on gene expression data (Table 1). A minor reduction in performance may be expected, as DNA methylation and gene expression do not correlate perfectly. When applying this signature to the gene expression data we obtained an area under the ROC curves of 0.870 (Fig. S1B). We then performed validation of the predictive signature in an independent cohort: the PROMIX clinical trial [29]. Since no DNA methylation data was available for this cohort, we applied the predictive “translated” gene expression signature to the ER‐positive subset generating a “translated DNA methylation score”, and observed an area under the ROC curve of 0.762 and with optimal cutoff selection we obtained a sensitivity of 60% and a specificity of 96% in the validation expression dataset (Fig. 2C).

When performing the analysis on both ER‐positive and ER‐negative tumors the prediction was poorer compared to ER‐positive only (area under the ROC curve of 0.639; Fig. S1C). Results could not be generated from the chemotherapy arm (too few patients with pCR), or ER‐negative patients in the combination arm (too few patients). Also, when applying the methodology directly on gene expression data, the prediction performance was inferior in both discovery and validation data (area under the ROC curve of 0.698 and 0.707, respectively; Fig. S1D,E).

### 3.2 A hybrid protein and DNA methylation classification improves prediction of treatment response from pre‐treatment biopsies

To assess the link between the identified DNA methylation signature and the published ViRP protein signature [13] we calculated the correlation between the obtained scores. The scores were highly correlated (Pearson correlation coefficient of 0.71; *P*‐value = 1.75e‐15; Fig. S1F). Further, to assess the complementarity of the two scores, we generated a hybrid score by averaging the scores from the two signatures. When applying the hybrid score on the validation set (PROMIX), we obtained a larger area under the ROC curve of 0.784, but the difference in AUC from the DNA methylation only score was not significant (DeLong test *P*‐value = 0.872). Selecting the optimal cutoff‐value we obtained a sensitivity of 60% and a specificity of 93% (Fig. 2D).

### 3.3 DNA methylation profiles after treatment with chemotherapy +/− bevacizumab

To identify alterations in DNA methylation between treatment timepoints common across samples, we performed paired *t*‐tests, and CpGs were considered significantly differentially methylated before and after treatment if the Benjamini–Hochberg corrected *P*‐value was smaller than 0.05 and absolute change in DNA methylation was larger than 0.1. In the combination arm 9460 CpGs were significantly differentially methylated between week 0 and week 12, 6940 CpGs were differentially methylated between week 12 and week 25, and 37 734 CpGs were differentially methylated between week 0 and week25. For the chemotherapy arm, fewer alterations were observed; 649 CpGs between week 0 and week 12, 311 CpGs between week 12 and week 25, and 13 164 between week 0 and week 25 (Table 2). Full lists of results are shown in Table S2A–F.

**Table 2:** Changes in DNA methylation during treatment with chemotherapy +/− bevacizumab. The number of significant CpGs with altered DNA methylation levels are shown. Significance was calculated using paired *t*‐tests, and results were considered significant if the Benjamini–Hochberg corrected *P*‐value was smaller than 0.05 and the absolute change in methylation was larger than 0.1. Number of samples in each analysis is shown in parentheses.

	Week 0 to week 12 (FEC)	Week 12 to week 25 (Taxanes)	Week 0 to week 25
Combination arm	9460 (*n* = 36)	6940 (*n* = 25)	37 734 (*n* = 35)
Chemotherapy arm	649 (*n* = 43)	311 (*n* = 33)	13 164 (*n* = 42)

To assess the function of the genes affected by treatment‐induced epigenetic alterations, we mapped CpGs to genes using standard Illumina annotation and performed GSEA of the genes with differential methylation (Table S3A). Several gene sets related to cell motility, cell adhesion and cell locomotion were enriched among the genes affected by both hypo‐ and hypermethylation, in both treatment arms, and between all timepoints. Neurogenesis and neuron functions were also enriched in several of the differential methylation analyses (Fig. S2).

### 3.4 Integration of DNA methylation and mRNA expression reveals biclusters with distinct biology

**Fig. 3:**
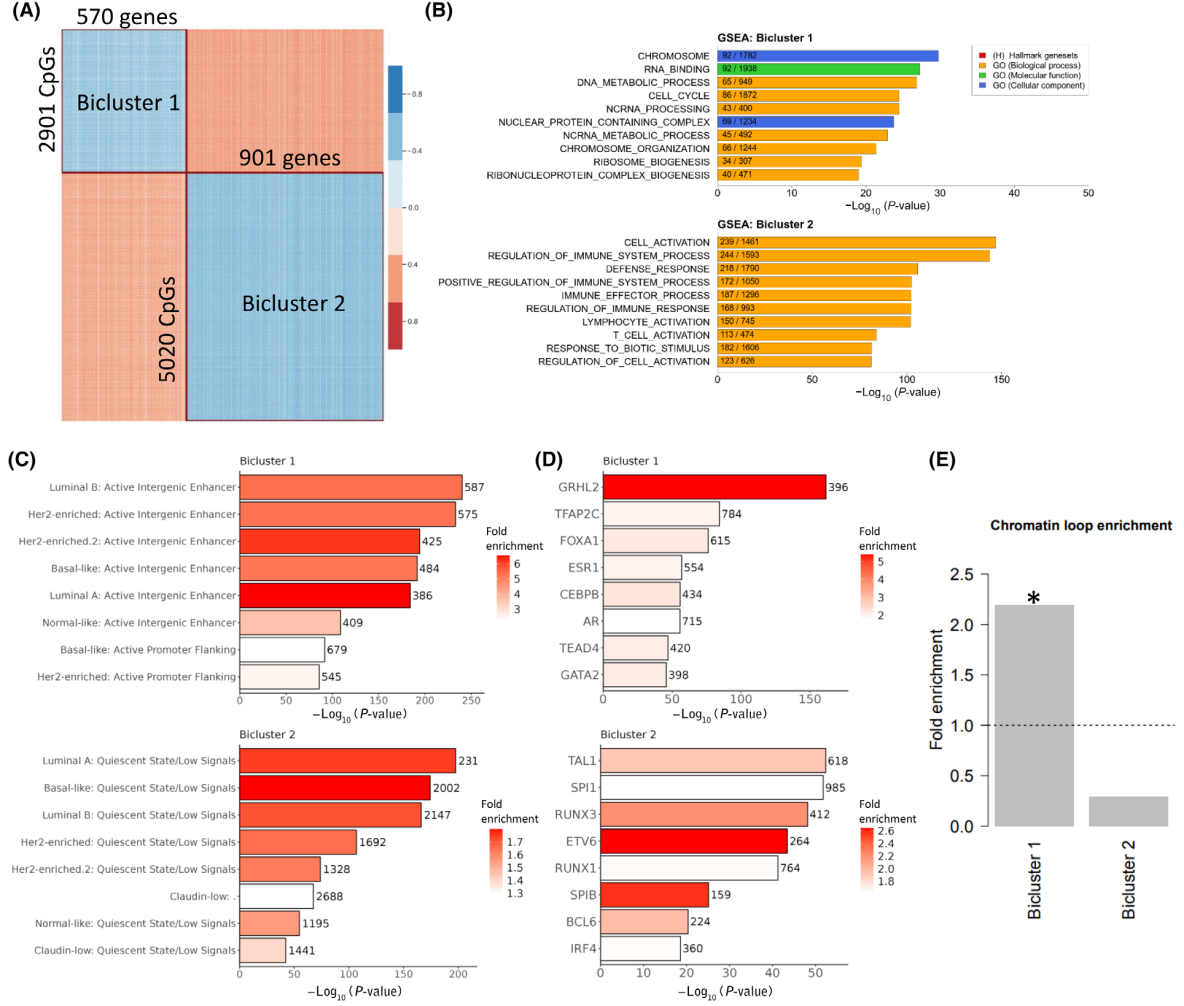
Identification of biclusters of CpGs and genes altered during treatment with chemotherapy +/− bevacizumab. (A) Spectral co‐clustering of correlation coefficients of delta expression‐methylation quantitative trait loci (emQTL) using *k*‐means identifies two biclusters; heatmap shows ordered rows and columns, where blue represents negative correlation and red represents positive correlation. (B) Gene set enrichment analysis (GSEA) of genes in the two biclusters. (C, D) functional epigenomic enrichment (ChromHMM) analysis of CpGs (C), and transcription factor binding region (UniBind) enrichment analysis (D) of CpGs in Bicluster 1 and Bicluster 2 (upper and lower panels, respectively). *P*‐values (*x*‐axis) are calculated using hypergeometric tests and fill color (C, D) denotes fold enrichment above what is expected by chance. (E) Fold enrichment of overlap between CpG‐gene pairs in biclusters and chromatin loops determined by chromatin interaction analysis by paired‐end tag sequencing (ChIA‐PET) in MCF7; statistical significance (Bicluster 1: *P* = 8.6e‐30(*); Bicluster 2: *P* = 1) was determined by hypergeometric test of observed loops compared to possible loops within each biclusters.

We have previously shown that genome‐wide associations (*in cis* and *in trans*) between DNA methylation and gene expression (emQTLs) are a powerful approach to identify transcriptional programs based on epigenetic modifications and TF activity, differentially regulated between patients [16, 43]. To identify such epigenetically regulated transcriptional programs involved in the response to therapy as well as patient group‐specific alterations in DNA methylation, we applied the emQTL approach to delta methylation and delta expression values before and after treatment with FEC +/− Bevacizumab (delta emQTL). The delta emQTL revealed 122 986 significant associations between delta methylation and delta expression (Bonferroni‐corrected *P*‐value < 0.05), involving 7921 CpGs and 1471 genes. Spectral co‐clustering was used to identify biclusters of CpGs and genes, representing CpGs and genes with enrichment of significant within‐bicluster associations (Fig. 3A). Two bicluster were chosen based on maximum silhouette scores (Fig. S3) and visual inspection of correlation and anticorrelation between CpGs and genes (Fig. 3A).

To assess the putative biological function of each bicluster, we performed GSEA on the gene list from each bicluster. Genes in Bicluster 1 were enriched in gene sets associated with chromosomal organization and cell cycle, while Bicluster 2 genes were enriched in gene sets related to immune‐related processes (Fig. 3B). To assess the epigenetic properties of the DNA surrounding the identified CpGs, we utilized ChromHMM segmentation data based on ChIP‐seq data of various histone modifications from different breast cancer cell lines covering the PAM50 subtypes. CpGs in Bicluster 1 were significantly enriched in active intergenic enhancer regions of cell lines representing all PAM50 subtypes (Fig. 3C upper panel), suggesting that methylation of these CpGs may contribute to epigenetic regulation through distal regulatory elements; CpGs in Bicluster 2, conversely, were enriched in regions with quiescent chromatin state in cancer cells (Fig. 3C lower panel).

We next investigated the TF binding regions surrounding the identified CpGs and TF‐DNA interaction data was obtained from UniBind [41]. Bicluster 1 CpGs were found enriched in binding regions of TFs including GRHL2, TFAP2C, FOXA1, ER, CEBPB and NR5A2, TFs known to be important in many cancer‐related functions such as proliferation, stemness and epithelial‐to‐mesenchymal transition (EMT) (Fig. 3D upper panel). Bicluster 2 CpGs were enriched in binding regions of TFs such as TAL1, SPI1, RUNX and ETV6, TFs known to be important in the development of the immune system (Fig. 3D lower panel).

The presence of chromatin loops between enhancers and promoters may suggest epigenetic regulation of genes, and we therefore investigated if the CpGs and genes within the two biclusters were located in chromatin loops of the ER‐positive breast cancer cell line MCF7 more often than expected by chance. CpGs and genes in Bicluster 1 was significantly enriched in chromatin loops (fold enrichment of 2.2; *P*‐value = 8.6e‐30), while CpGs and genes in Bicluster 2 were not enriched (Fig. 3E). These results further support an epigenetic regulation of genes in Bicluster 1.

### 3.5 Alterations in DNA methylation with concomitant changes in gene expression associated with treatment response

**Fig. 4:**
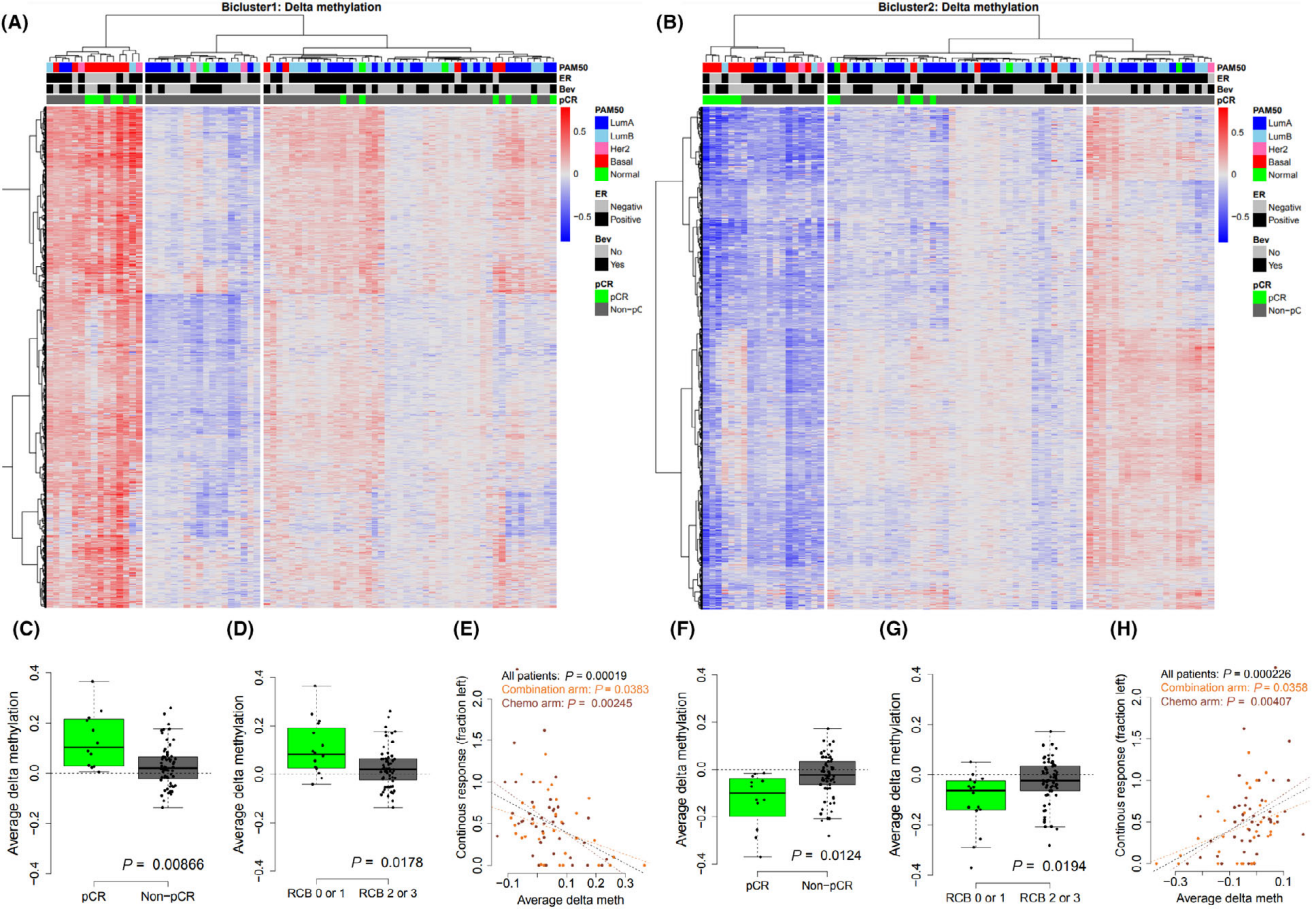
Delta DNA methylation of the identified biclusters after treatment with chemotherapy +/− bevacizumab and association to treatment response. Hierarchical clustering and heatmap of delta methylation values in Bicluster 1 (A) and Bicluster 2 (B); red is gain of methylation and blue is loss of methylation. Three patient clusters were in identified in both dendrograms. Patients (columns) are annotated with PAM50 subtype, estrogen receptor (ER) status, administration of bevacizumab and whether the patient achieved pathological complete response (pCR). (C–D, F–G) Boxplot showing average delta methylation of Bicluster 1 and 2 CpGs, respectively, plotted against achievement of pCR or residual cancer burden (RCB). Black dashed line denotes no change in methylation. Statistical significance is calculated using a *t*‐test. (E, H) Correlation between average delta methylation of Bicluster 1 and 2 CpGs, respectively, and the fraction of tumor left for all patients (black), combination arm (orange) and chemo arm (brown). Dashed lines denote linear regressions between average delta methylation and response. Statistical significance is calculated using Pearson correlation test.

To assess the molecular alterations during treatment and the relation to treatment response, we performed hierarchical clustering of the delta methylation and delta expression levels of CpGs and genes in the two identified biclusters. For Bicluster 1, patients were divided into three main clusters characterized by strong gain in methylation, moderate gain in methylation and loss of methylation (Fig. 4A). Tumors in the clusters that showed strong gain of methylation were predominantly ER‐negative and Basal‐like, while tumors in the two other clusters were mainly ER positive and Luminal A or Luminal B; both Luminal A and Luminal B tumors were evenly distributed between these clusters. Patients with high gain of methylation showed high rates of pCR (6/15), patients with moderate gain of methylation showed moderate rates of pCR (6/46) while patients with loss of methylation included no patients achieving pCR (0/17; Fig. 4A). Delta methylation values for Bicluster 2 CpGs showed an inverse pattern compared to Bicluster 1, as the ER‐negative (Basal‐like) tumors were enriched in the cluster with strong loss of methylation, while the ER‐positive patients were divided into two clusters showing either moderate loss of methylation or gain of methylation. Luminal A and Luminal B tumors were evenly distributed between the two ER‐positive clusters. The cluster with moderate loss of methylation contained more patients with pCR compared to the cluster with gain of methylation (Fig. 4B).

To further confirm the association between delta methylation and response to treatment, we averaged the delta methylation levels for all identified CpGs in each bicluster and compared this score against response to treatment (pCR or RCB). Patients achieving pCR or RCB 0 (pCR) or RCB 1 showed increase in delta methylation of Bicluster 1 CpGs and loss of methylation in Bicluster 2 CpGs (*P*‐values < 0.05; Fig. 4C–D,F–G). Further, we performed correlation analyses between the average delta methylation score and the fraction of tumor left after treatment, and we observed a significant correlation for both Bicluster 1‐ and Bicluster 2‐CpGs for all samples and for each arm independently (all *P*‐values < 0.05; Fig. 4E,H). For Bicluster 1 CpGs gain of methylation was associated to better response, and for Bicluster 2 CpGs loss of methylation was associated to better response.

**Fig. 5:**
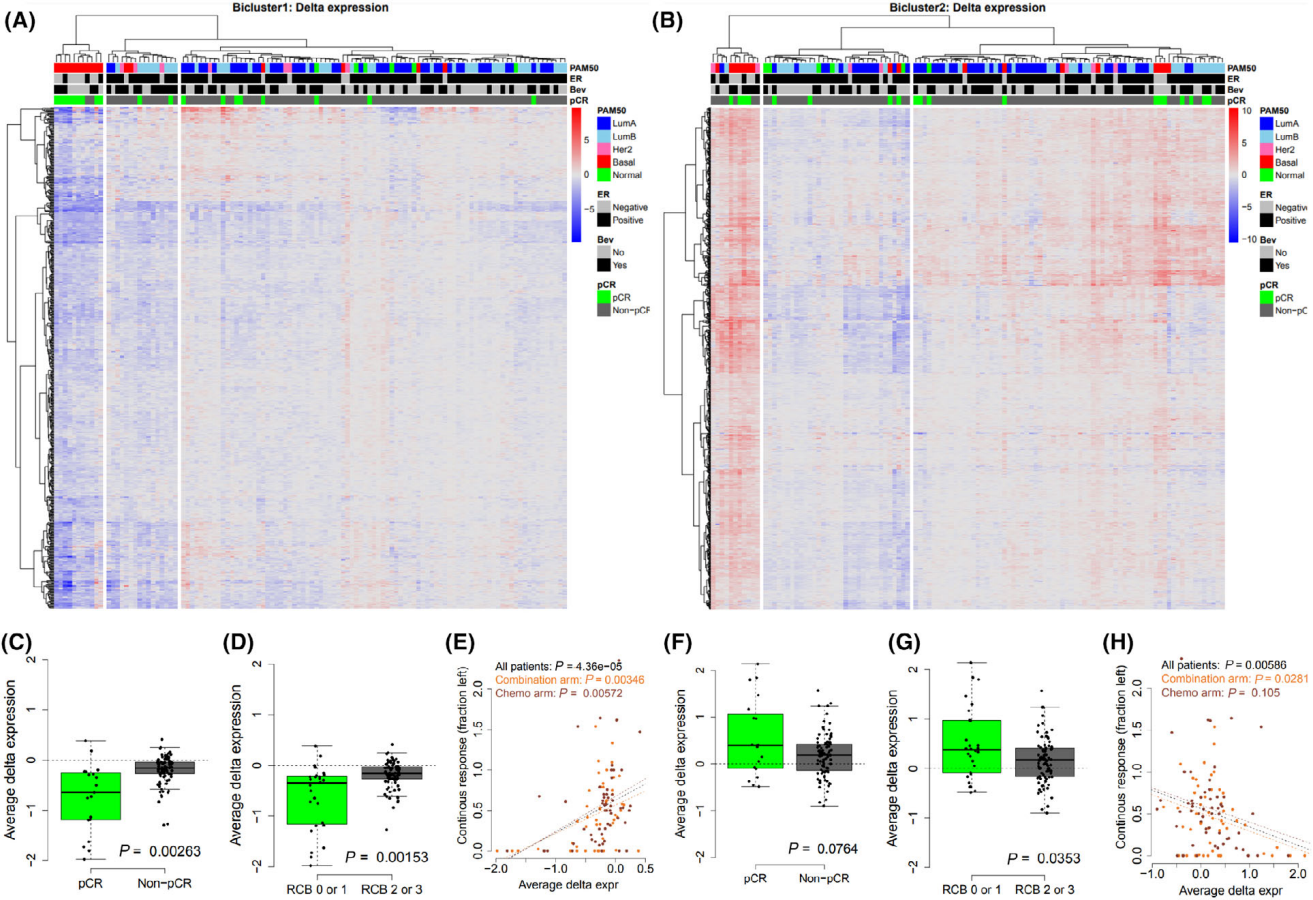
Delta gene expression of the identified biclusters after treatment with chemotherapy +/− bevacizumab and association to treatment response. (A, B) Hierarchical clustering and heatmap of delta expression values; red is gain of expression and blue is loss of expression. Three patient clusters were in identified in both dendrograms. Patients (columns) are annotated with PAM50 subtype, estrogen receptor (ER) status, administration of bevacizumab and whether the patient achieved pathological complete response (pCR). (C–D, F–G) Boxplot showing average delta expression of Bicluster 1 and 2 genes, respectively, plotted against achievement of pCR or residual cancer burden (RCB). Black dashed line denotes no change in expression. Statistical significance is calculated using a *t*‐test. (E, H) Correlation between average delta expression of Bicluster 1 and 2 genes, respectively, and fraction of tumor left for all patients (black), combination arm (orange) and chemo arm (brown). Dashed lines denote linear regressions between average delta expression and response. Statistical significance is calculated using Pearson correlation test.

The alterations in DNA methylation were accompanied by alterations in gene expression (Fig. 5A,B). For genes in Bicluster 1 decreased gene expression was associated with better response (Fig. 5C–E), and for genes in Bicluster 2 increased expression was associated with better response (Fig. 5F–H).

We performed validation in an independent patient cohort (Doxo/Fumi) where patients with late‐stage breast tumors received neoadjuvant treatment with either Doxorubicin or Fluorouracil (5FU) and mitomycin C. In concordance with the observed alterations in DNA methylation from the present patient cohort (NeoAva; Fig. 4A,B), we observed patient clusters with both gain and loss of DNA methylation for both Bicluster 1 and Bicluster 2 CpGs (Fig. S4). The ER status of tumors was more evenly distributed across the clusters with gain or loss of methylation, perhaps highlighting that the patients in the Doxo/Fumi trial responded less to the treatment [30, 31].

### 3.6 Cancer cell‐specific profiles of DNA methylation

Since many of the patients achieved good response to the treatment, it was likely that the observed treatment‐induced changes in DNA methylation could be confounded by a higher proportion of non‐tumor cells present in the samples taken at week 12 and week 25. To determine alterations in DNA methylation specific to the cancer cells, we developed a method to correct the measured DNA methylation values to represent only cancer cells (see Section 2).

To identify cancer cell‐specific alterations in DNA methylation between treatment timepoints common across samples, we repeated the paired analyses between timepoints using the tumor purity‐corrected DNA methylation levels. In the combination arm, 378 CpGs were differentially methylated between week 0 and week 12 and 5107 CpGs were differentially methylated between week 12 and week 25. Fewer CpGs were differentially methylated in the chemotherapy arm, 46 and 58 between week 0 and week 12, and week 12 and week 25, respectively (Table 3; Full lists of results are shown in Table S2G–L). To assess the function of the implicated genes in the tumor cells, we mapped CpGs to genes using standard Illumina annotation and performed GSEA of the genes with differential methylation (Table S3B). From week 0 to week 12 in the combination arm the hypomethylated genes were enriched in several gene sets related to adhesion, while the hypermethylated genes were enriched in gene sets related to locomotion, migration and cytoskeleton organization (Fig. S5). From week 12 to week 25 in the combination arm both hypomethylated and hypermethylated genes were enriched in gene sets related to adhesion and locomotion (Fig. S5). In the chemotherapy arm, too few genes were differentially methylated to perform GSEA. From week 0 to week 25 in the combination arm, differentially methylated genes were also enriched in gene sets related to adhesion, and in the chemotherapy arm differentially methylated genes were enriched in gene sets related to abnormal cardiovascular physiology and immune functions.

**Table 3:** Changes in tumor purity‐corrected DNA methylation during treatment with chemotherapy +/− bevacizumab. The number of significant CpGs with altered DNA methylation levels are shown. Significance was calculated using paired *t*‐tests, and results were considered significant if the Benjamini–Hochberg corrected *P*‐value was smaller than 0.05 and the absolute change in methylation was larger than 0.1. Number of samples in each analysis is shown in parentheses.

	Week 0 to week 12 (FEC)	Week 12 to week 25 (Taxanes)	Week 0 to week 25
Combination arm	378 (*n* = 35)	5107 (*n* = 25)	3190 (*n* = 34)
Chemotherapy arm	46 (*n* = 37)	58 (*n* = 29)	386 (*n* = 41)

To assess whether the delta emQTL results were influenced by lower proportion of tumor cells in the treated biopsies, we performed hierarchal clustering also on the tumor purity‐adjusted delta DNA methylation levels. Delta methylation levels of Bicluster 1 CpGs showed concordant patterns (compared to non‐adjusted data) across tumors, with two clusters showing gain of methylation and one showing loss of methylation (Fig. S6A), suggesting that the altered methylation of these CpGs were likely caused by alterations in the cancer cells or altered composition of cancer cell subclones. Delta methylation levels of Bicluster 2 CpGs also showed loss of methylation for the ER‐negative tumors, but the pattern for ER‐positive tumors was less clear (Fig. S6B), again implying that methylation levels of these CpGs were more influenced by non‐cancer cells such as immune cells. When comparing average tumor purity‐adjusted delta methylation to response, we observed that the differences in average methylation was still (borderline) significant for Bicluster 1 CpGs (pCR *P*‐value = 0.052; RCB *P*‐value = 0.032; Fig. S6C,D), but not significant for CpGs in Bicluster 2 (pCR *P*‐value = 0.168; RCB *P*‐value = 0.081; Fig. S6E,F).

To confirm that the predictive signature was not confounded by tumor purity, we applied the stable model (Table 1) on tumor purity‐adjusted DNA methylation data and we achieved a perfect prediction performance (AUC = 1), in concordance with the results obtained using non‐adjusted DNA methylation data.

## 4 Discussion

Here we present DNA methylation profiles of fresh‐frozen tumor biopsies taken before, during and after treatment from patients receiving neoadjuvant chemotherapy with or without bevacizumab. We report that DNA methylation at enhancer CpGs related to cell cycle, estrogen response and immune infiltration can predict response to the combination of chemotherapy and bevacizumab, and we validated this in an independent patient cohort from a similar clinical trial [29]. When combining the DNA methylation score with the previously published ViRP proteomic score [13] for the PROMIX data set, we observed a trend indicating increased prediction performance. We have previously reported that administration of bevacizumab in addition to chemotherapy may confer improved response for patients [11], and here we show that tumors receiving the combination treatment underwent more extensive epigenetic remodeling than tumors receiving only chemotherapy both in bulk and when corrected for tumor purity. We then performed integrative emQTL analysis on alterations in DNA methylation and gene expression levels, showing that the epigenetic alterations that occur during treatment are different between responders and non‐responders and that these differences may be explained by the proliferation‐EMT axis through the activity of GRHL2 and other TFs.

The use of bevacizumab plus chemotherapy as first‐line treatment in patients with locally advanced or metastatic breast cancer has shown improved response and clinical benefit in the present study (NeoAva) [11] and others [6, 9, 10, 44, 45]. Here, we present a predictive biomarker for selection of patients with ER‐positive HER2‐negative tumors that have a high probability of improved response by the addition of bevacizumab. Since ER‐positive and ER‐negative tumors are different with regards to drivers of proliferation, it is an advantage that the predictive signature is trained and validated on ER‐positive tumors specifically. Taken together, these results illustrate the clinical benefit of bevacizumab if administered to the correct patients. A limitation of the study is the lack of available follow‐up data to assess whether the response to the neoadjuvant treatment translates into increased relapse‐free survival (RFS), progression‐free survival (PFS) or overall survival (OS). However, it has previously been shown that RCB (both continuous and categorical) translates into better RFS [46].

Preselection of CpGs for identification of the predictive signature was performed based on the results in Ankill et al. [17], where we show that 44 263 CpGs capture important biological variation in breast cancer involving estrogen signaling, non‐estrogen‐controlled regulation of proliferation, immune infiltration and fibroblast infiltration. The majority of CpGs in the identified predictive signature were associated to regulation of the cell cycle, showing that epigenetic marks related to proliferation can be predictive of response, which is in concordance with previous studies [18, 26]. The NeoAva (discovery cohort) and PROMIX (validation cohort) are similar in patient characteristics (Table S1), but differ slightly in treatment regimens. The patients in NeoAva got FEC followed by docetaxel, while the patients in PROMIX got epirubicin together with docetaxel. As we used an internal cross‐validation (leave‐one‐out) approach, the prediction results from NeoAva could also be interpreted without external validation because each prediction is not using the patient's own data for training. In addition, since the predictive signature also predicts response in patients with a different chemotherapy regimen, the results suggest that the identified signature captures the effect of adding bevacizumab.

The delta emQTL approach has the advantage of identifying concomitant alteration in DNA methylation and gene expression that are different between patients, which allowed us to identify CpGs and genes whose alterations were associated to response. We identified two biclusters of CpGs and genes with negative within‐bicluster correlations, one with enrichment in enhancers and TFs important in functions such as proliferation, stemness and EMT, and one with enrichment in immune‐related TFs, both of which were (oppositely) associated with response. We validated the profiles of alterations in DNA methylation during chemotherapy in an independent patient cohort treated with doxorubicin or a combination of 5‐FU and mitomycin, but we did not validate the association to response; the lack of validation to response may be due to that the two patient cohorts were collected at different time periods, and that the tumors in the validation cohort were larger, and the response rates were a lot poorer overall.

The association between treatment response and alteration of methylation in enhancer CpGs enriched in GRHL2 binding regions with concomitant changes of proliferation‐related genes (Bicluster 1) suggests that proliferative cells with high activity of GRHL2 are killed by the treatment leading to an observed gain of methylation and loss of expression (Figs 4A and 5A). GRHL2 (Grainyhead‐like‐2) is known to promote proliferation and suppress EMT by repressing ZEB1 expression and inhibiting TGF‐β signaling [47], and to suppress the emergence of a stem cell phenotype [48]. We observe that gain of GRHL2 activity through loss of enhancer methylation with following increase in proliferation‐related genes is characteristic of non‐responders, suggesting that non‐responding tumor cells can gain proliferative potential during treatment stress. For Bicluster 2, the observed alterations in methylation and expression suggest that eradication of tumor cells lead to a relative increase in non‐tumor cells (e.g., immune cells) in the tumors that respond well to treatment, which in turn is reflected in the loss of methylation at enhancers carrying TF binding regions of TFs related to immune development and increase in expression of immune‐related genes.

In this study we present a method for adjusting DNA methylation levels to reflect cancer cells only (cancer cell‐specific DNA methylation) when tumor purity estimates are available. In our results, analyses with cancer cell‐specific data showed that the predictive signature in pre‐treated biopsies was not affected by tumor purity. From the delta emQTL analysis, Bicluster 1 showed similar results with original DNA methylation and cancer cell‐specific data, while Bicluster 2 showed less variation and association to response in the cancer cell‐specific methylation data, suggesting that Bicluster 2 was more driven by changes in tumor purity. In the paired *t*‐test analyses, we observed fewer differentially methylated CpGs, but the genes affected were mainly enriched in the same biological functions (i.e., adhesion, locomotion etc.; Figs S2 and S5). Conversely, the neurogenesis and neuron function gene sets were less enriched in the cancer cell‐specific methylation data, suggesting that these results may be caused by changes in tumor purity (Figs S2 and S5). The method for tumor purity‐corrected DNA methylation uses a linear regression approach where values are replaced with the residual of the regression model. This approach is commonly used when correcting gene expression values for unwanted covariates such as RIN value. In this case, the approach assumes that the methylation level of each CpG is equal in all non‐tumor cells, and that deviations from “expected” (i.e., the regression line) is attributed to tumor cells. Since DNA methylation alterations in breast tumors are large and frequent, this assumption is reasonable, but not true for all CpGs. Nonetheless, the method is useful when studying tumors where tumor purity is an important and unwanted confounding factor.

## 5 Conclusion

Here we report that DNA methylation at enhancer CpGs related to cell cycle regulation can predict response to the treatment with chemotherapy and bevacizumab for ER‐positive patients, and we validated this observation in an independent patient cohort. The DNA methylation scores were highly correlated with the scores from the protein signature (ViRP), and combining the scores resulted in a slight increase in the prediction performance. We also show that tumors receiving the combination treatment underwent more extensive epigenetic alterations than tumors receiving only chemotherapy. Finally, we perform the integrative emQTL analysis on alterations in DNA methylation and gene expression levels, showing that the epigenetic alterations occurring during treatment differs between responders and non‐responders and that these differences may be explained by the proliferation‐EMT axis through the activity of the transcription factor GRHL2. Taken together, these results illustrate the importance of predictive markers to identify patients with clinical benefit of the addition of bevacizumab to chemotherapy.

## Conflict of interest

Mads Haugland Haugen and Olav Engebraaten: Patent application submitted for a nine‐protein/gene panel predicting response to anti‐VEGF therapies in combination with chemotherapy. No other potential conflicts of interest were reported.

## Author contributions

TF designed the study, participated in data generation, performed data analysis, data interpretation and wrote the manuscript. MHH performed data analysis, data interpretation and wrote the manuscript. JA participated in data analysis. LS‐P participated in data generation. A‐LB‐D participated in design of the study. IH and TH supplied the data from the validation cohort. JT generated the DNA methylation data. OE initiated and led the clinical trial and participated in designing the study. VNK designed the study. All co‐authors participated in data interpretation, revised the manuscript, and approved the final version of the manuscript.

### Peer review

The peer review history for this article is available at https://www.webofscience.com/api/gateway/wos/peer‐review/10.1002/1878‐0261.13656.

## Supporting information


**Fig. S1.** Prediction of response to chemotherapy and bevacizumab.
**Fig. S2.** Characterization of genes affected by treatment‐induced epigenetic alterations.
**Fig. S3.** Average silhouette score for different number of biclusters.
**Fig. S4.** Validation of delta emQTLs in an independent clinical trial. Delta DNA methylation of the identified biclusters after treatment with Doxorubicin or Fluorouracil (5FU) and mitomycin C.
**Fig. S5.** Characterization of genes affected by treatment‐induced estimated cancer‐specific DNA methylation alterations.
**Fig. S6.** Tumor purity‐adjusted delta DNA methylation of the identified biclusters after treatment with FEC +/− bevacizumab.

**Table S1.** Patient characteristics.

**Table S2.** Differentially methylated CpGs during treatment.

**Table S3.** Genes affected by differential DNA methylation.

## Data Availability

The raw and normalized DNA methylation data for NeoAva are available in GEO with accession number GSE207460. The NeoAva gene expression data are available in the ArrayExpress database (http://www.ebi.ac.uk/arrayexpress) under accession number E‐MTAB‐4439. The CNA data are available at the European Genome‐phenome Archive under accession number EGAS00001003287. The PROMIX gene expression data is available through GEO with accession number GSE87455.
