# Supplementary material for: An integrated omics approach highlights how epigenetic events can explain and predict response to neoadjuvant chemotherapy and bevacizumab in breast cancer
Source: Mol Oncol. 2024 Apr 26;18(8):2042–59. doi: 10.1002/1878-0261.13656 (PMC11306529; doi:10.1002/1878-0261.13656)
Supplement: Supplementary file 1 — Fig. S1. Prediction of response to chemotherapy and bevacizumab. Fig. S2. Characterization of genes affected by treatment‐induced epigenetic alterations. Fig. S3. Average silhouette score for different number of biclusters. Fig. S4. Validation of delta emQTLs in an independent clinical trial. Delta DNA methylation of the identified biclusters after treatment with Doxorubicin or Fluorouracil (5FU) and mitomycin C. Fig. S5. Characterization of genes affected by treatment‐induced estimated cancer‐specific DNA methylation alterations. Fig. S6. Tumor purity‐adjusted delta DNA methylation of the identified biclusters after treatment with FEC +/− bevacizumab. [file MOL2-18-2042-s004.pdf]

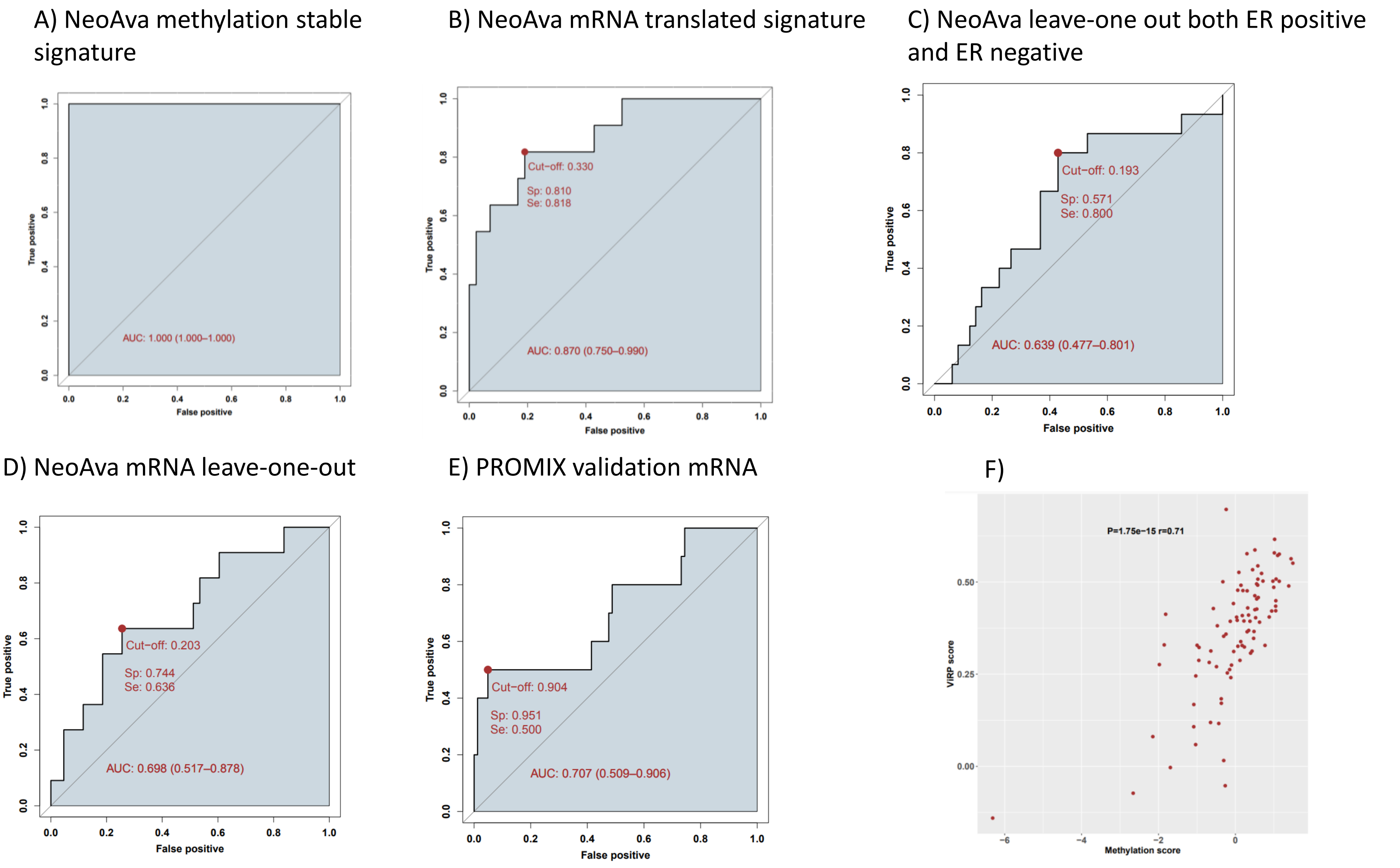

Supplementary Figure 1: Prediction of response to chemotherapy and bevacizumab. A) ROC curves for the identified stable predictive DNA methylation signature in NeoAva; B) ROC curve for the "translated" DNA methylation to gene expression signature in NeoAva; C) ROC curve for the leave-one-out cross-validation using methylation for both ER positive and ER negative; D) ROC curve for leave-one-out cross-validation using gene expression for ER positive; E) Validation of the gene expression signature (from D) in PROMIX; F) Correlation between the DNA methylation score and the ViRP protein score.

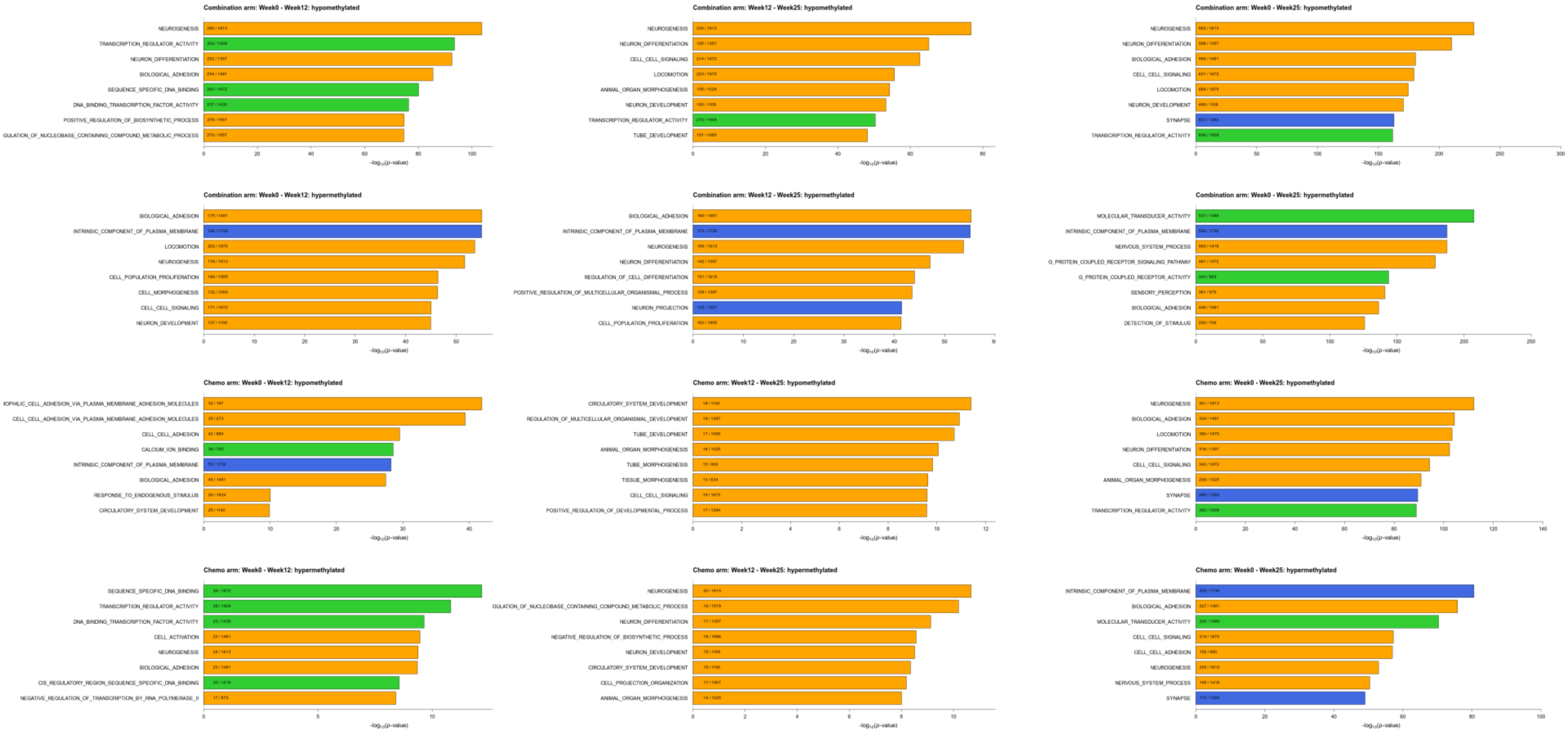

Supplementary Figure 2: Characterization of genes affected by treatment-induced epigenetic alterations. Gene set enrichment analysis (Hallmark and C5) of genes associated with differentially methylated CpGs. The length of the bars shows the log-transformed p-values obtained by hypergeometric testing. Red: Hallmark; blue: GO cellular compartment; orange: GO biological process; green: GO molecular function.

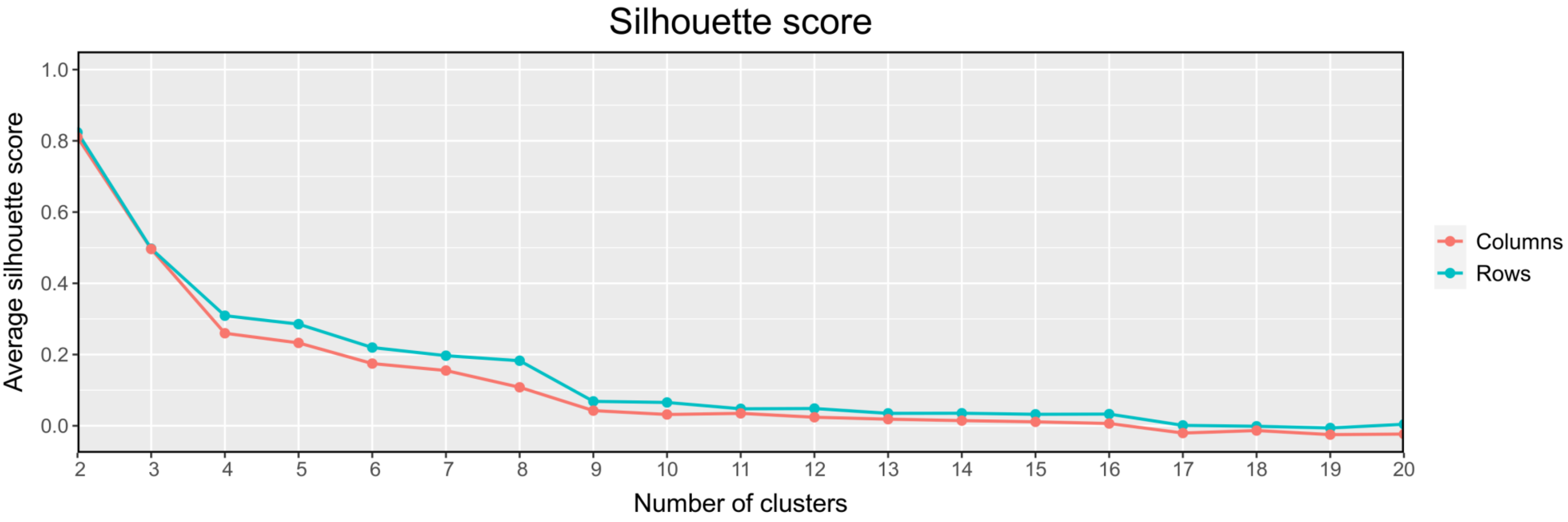

Supplementary Figure 3: Average silhouette score for different number of biclusters. Line chart showing the average silhouette scores for the biclusters rows and columns when the number of biclusters were set to be a number between 2 and 20.

A) B)

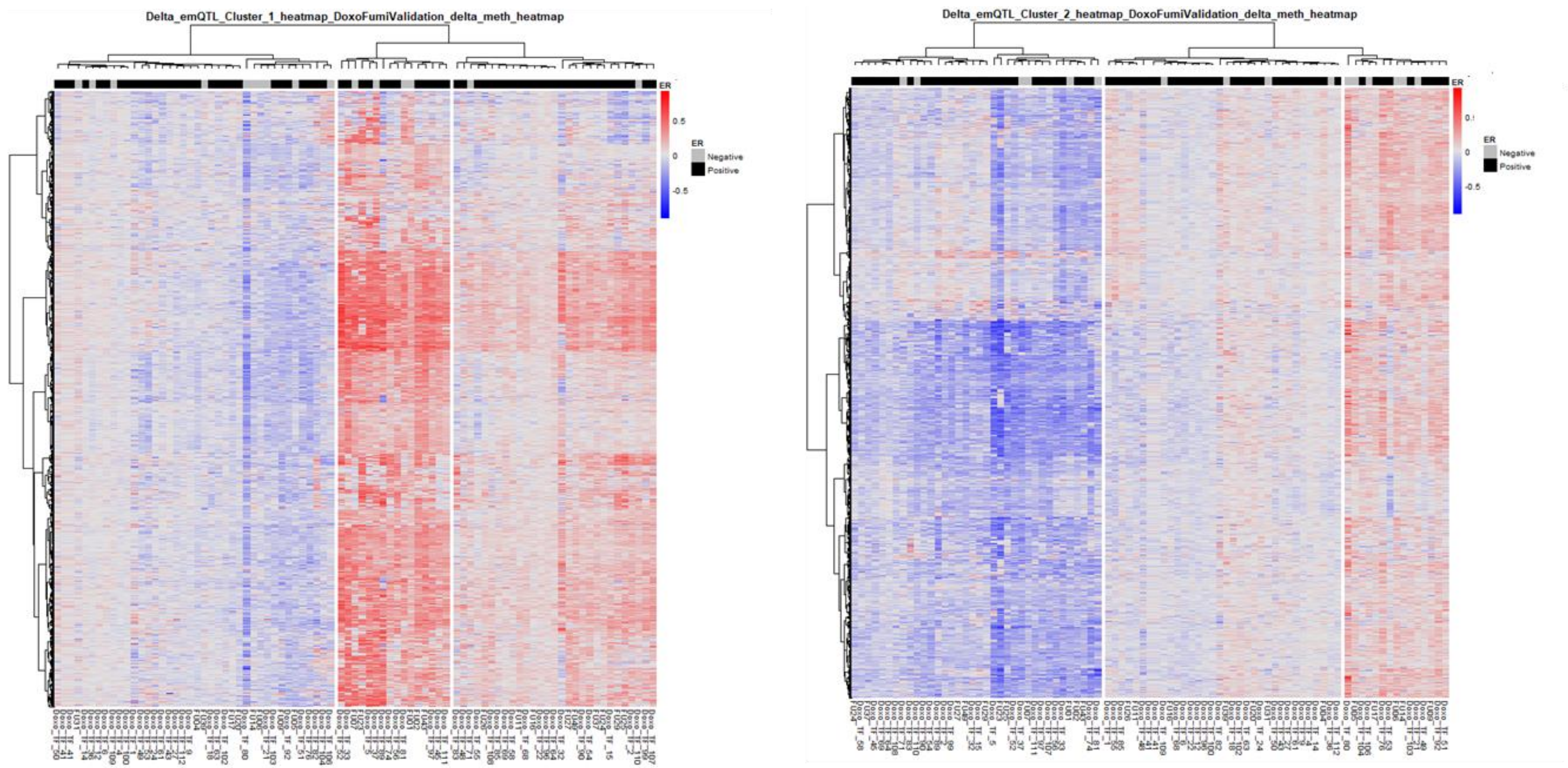

Supplementary Figure 4: Validation of delta emQTLs in an independent clinical trial. Delta DNA methylation of the identified biclusters after treatment with Doxorubicin or Fluorouracil (5FU) and mitomycin C. Hierarchical clustering and heatmap of delta methylation values of bicluster 1 (A) and bicluster 2 (B); red is gain of methylation and blue is loss of methylation. Patients (columns) are annotated with ER status.

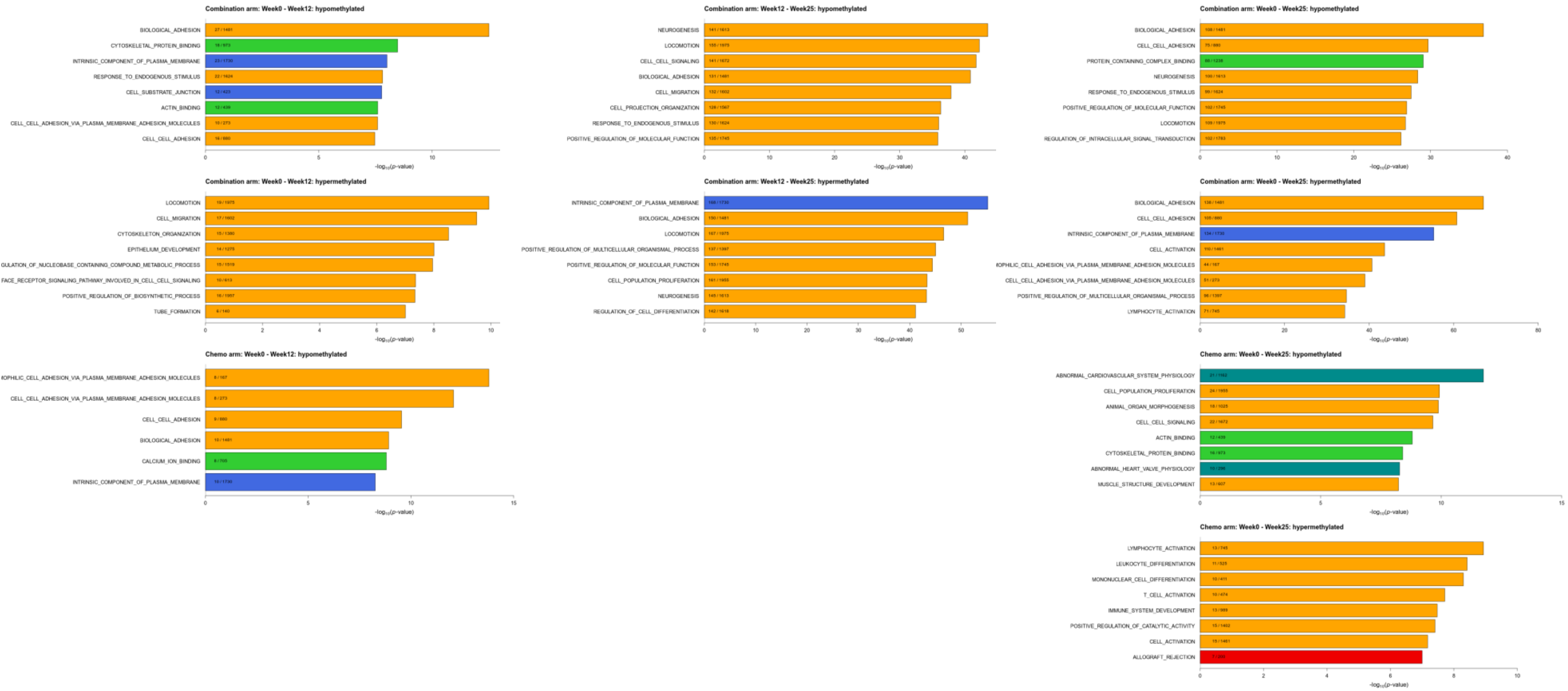

Supplementary Figure 5: Characterization of genes affected by treatment-induced estimated cancer-specific DNA methylation alterations. Gene set enrichment analysis (Hallmark and C5) of genes associated with differentially methylated CpGs. The length of the bars shows the log-transformed p-values obtained by hypergeometric testing. Red: Hallmark; blue: GO cellular compartment; orange: GO biological process; green: GO molecular function. Because of too few differentially methylated CpGs, no results were available between week 0 and week 12 for the chemo arm for the hypermethylated CpGs, or between week 12 and week 25 for the chemo arm.

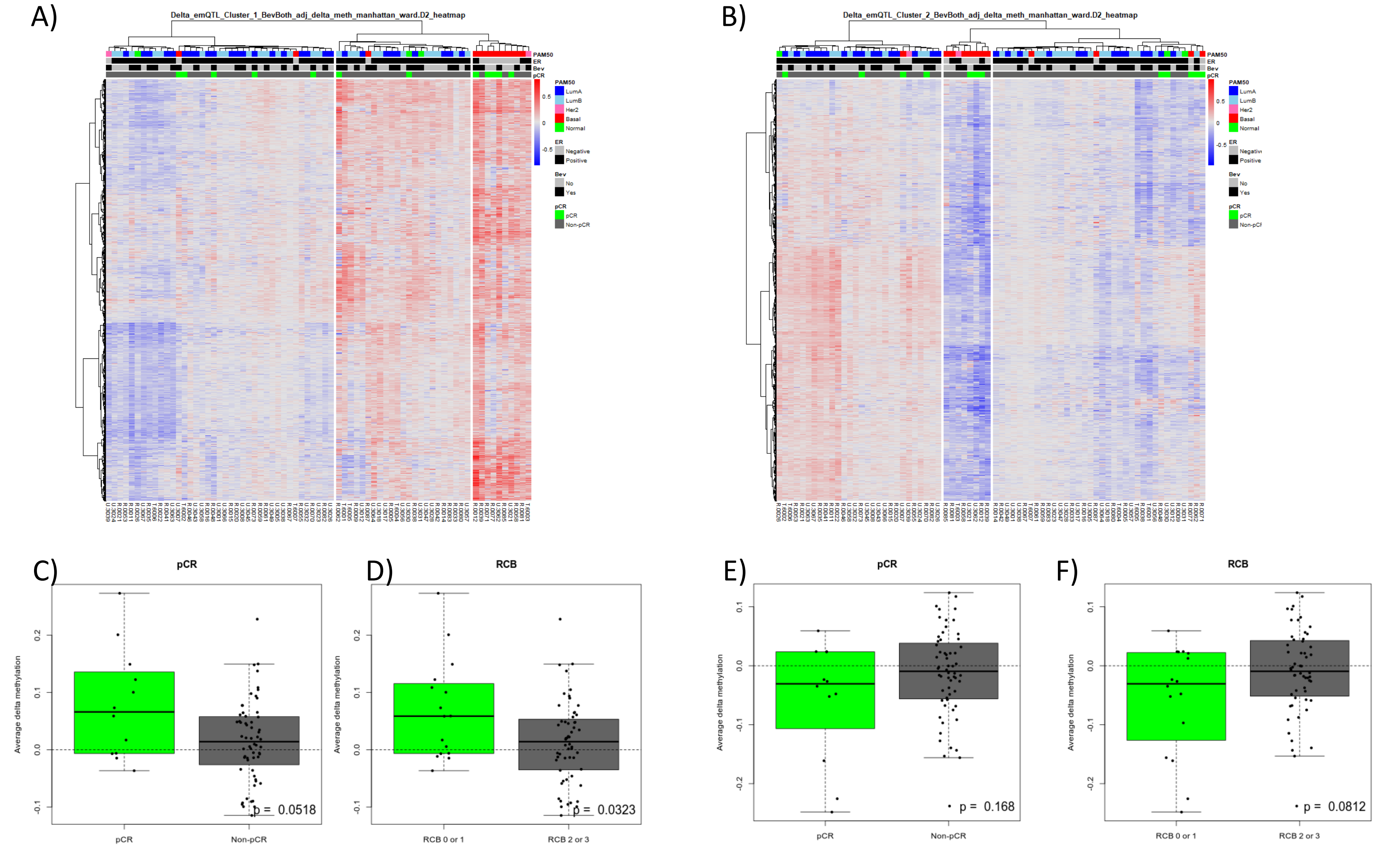

Supplementary Figure 6: Tumor purity-adjusted delta DNA methylation of the identified biclusters after treatment with FEC +/- bevacizumab. Hierarchical clustering and heatmap of delta methylation values of bicluster 1 (A) and bicluster 2 (B); red is gain of methylation and blue is loss of methylation. Patients (columns) are annotated with PAM50 subtype, ER status, administration of bevacizumab and whether the patient achieved pCR. C-D and E-F) Boxplot showing average tumor purity-adjusted delta methylation of Bicluster 1 and 2 CpGs, respectively, plotted against achievement of pCR or RCB. Black dashed line denotes no change in methylation. Statistical significance is calculated using t-test.
